# Surgeon-General of the Navy

**Published:** 1878-12

**Authors:** 


### Surgeon-General of the Navy.

Unusual interest has been felt by the medical corps of the navy in the appointment of a "chief of the Bureau of Medicine and Surgery" in the Navy department at Washington; the late incumbent, Surgeon William Grier, having recently retired by reason of age.

The Bureau of Medicine and Surgery, next that of Docks and Yards, is the most important of the departmental bureaus, and an appointment as its chief carries with the office the relative rank and pay of a commodore, and the title of "Surgeon-General of the Navy." With but a single exception it has always been given to the senior surgeon on the active list, and the importance attached to the present selection of this official has grown out of the fact that the secretary of the navy, on account of the comparatively brief time remaining before the officer whose name headed the list would be retired by reason of age, has seemed disposed to disregard precedent, and appoint to the vacant position a junior member of the medical corps. Great satisfaction is therefore felt that the head of the department has adhered to a custom, the non-observance of which would have had so demoralizing an influence.

Medical Director J. Winthrop Taylor, upon whom the surgeon-generalship has been conferred, was born in New York, in 1817. Graduating from Princeton College, and from the medical department of the University of Pennsylvania, he was appointed to the United States navy from the state of New Jersey in 1838. He has spent seven years and sixteen months at sea, an amount of service which has been equaled by but two other medical officers of the active list. He was in active duty throughout the Mexican war and the rebellion. He accompanied the expedition sent to bring the filibuster General Walker away from Nicaragua, and commanded in person the detail which conducted the prisoner by a long and perilous journey to the coast. He was surgeon of the Pensacola, when she ran the blockade of rebel forts erected along the Potomac River for her destruction, and was on duty in the same ship when, under Farragut, she passed the Mississippi forts, and assisted in the capture of New Orleans.

Surgeon-General Taylor has resided for many years in Boston, having been repeatedly attached to the Navy Yard, the Rendezvous, and the receiving ship at Charlestown, and to the Naval Hospital at Chelsea. This crowning honor of an active and unblemished service of forty years will give pleasure to a large circle of friends, by whom he is well known for his urbanity and his high standing as an officer and a gentleman.--*Boston Journal, October* 31, 1878.

